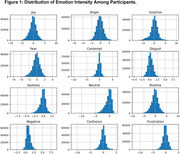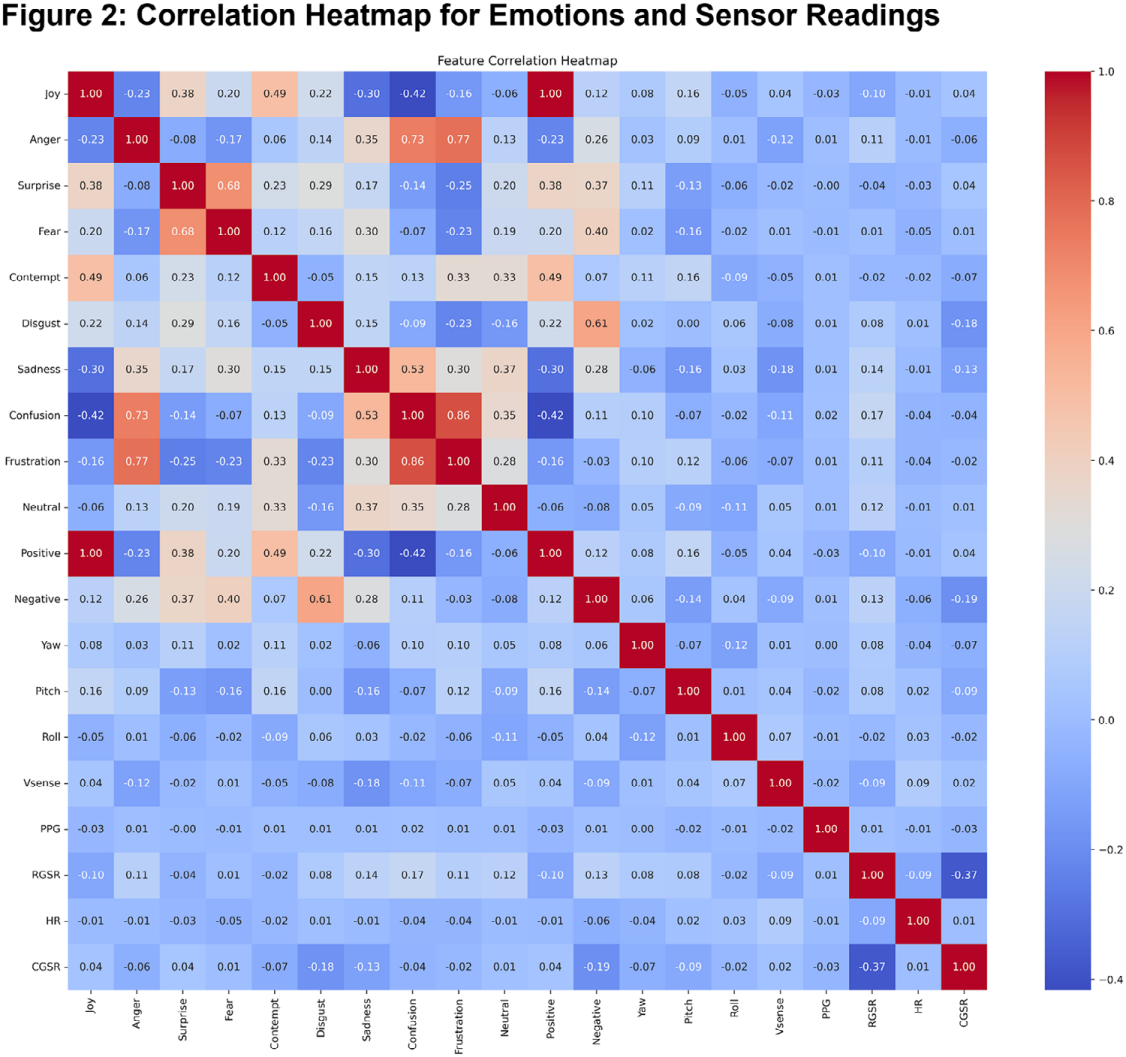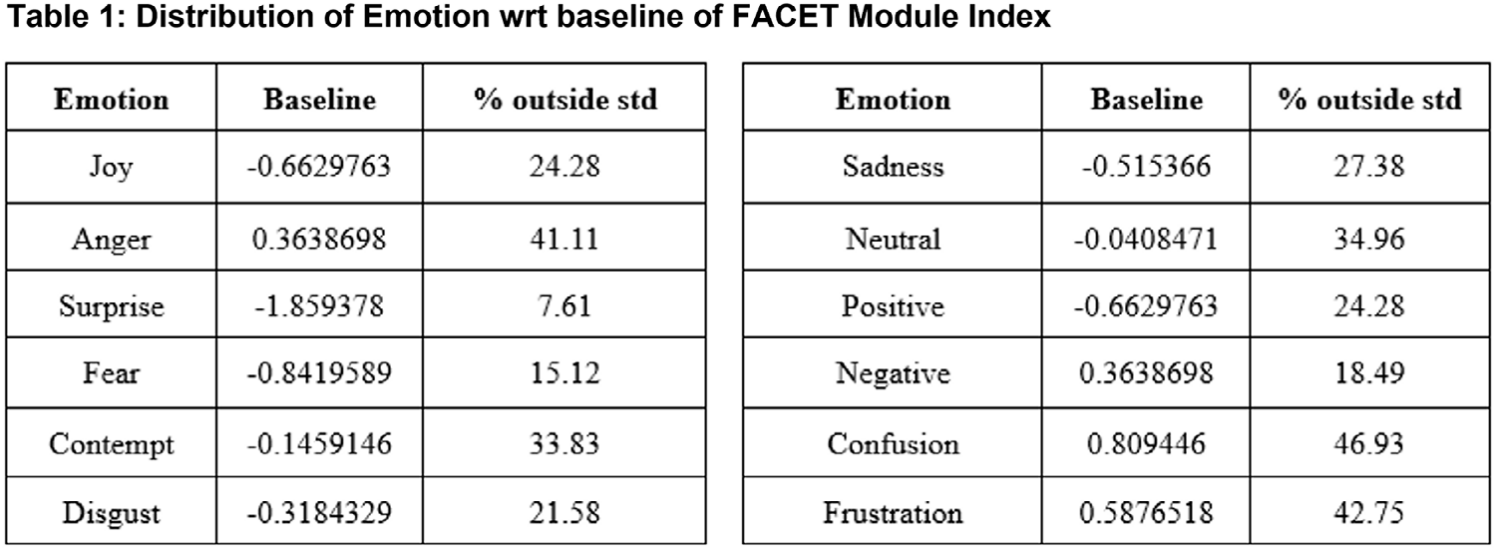# Exploring the Feasibility of Wearable Sensors for Emotion Detection of Older Adults

**DOI:** 10.1002/alz70857_105823

**Published:** 2025-12-25

**Authors:** Md Saif Hassan Onim, Himanshu Thapliyal

**Affiliations:** ^1^ University of Tennessee, Knoxville, TN, USA

## Abstract

**Background:**

Emotions play a crucial role in shaping human interactions, decision‐making, and overall well‐being. Emotional assessment is particularly critical for older adults and individuals with Alzheimer's Disease and Alzheimer's Disease Related Dementias (AD/ADRD). Facial expressions are the gold standard of emotion detection in older adults. Due to the applicability in real‐world settings, emotion detection through wearable sensors will be of significant interest. We aim to capture emotional states and their underlying correlation with wearable sensors, considering facial expression as the gold standard. Our analysis can help develop an effective wearable emotion detection system for older adults and people with AD/ADRD.

**Method:**

The study involved 50 older adults (range: 60‐75 years). They wore wearable sensors that captured physiological data. A facial recognition system analyzed their expressions in real time. Key features include Participant age, and quantified emotions such as Joy, Anger, Surprise, Fear, Contempt, Disgust, and Sadness, derived from facial expression analysis. Physiological signals captured using wearable devices include Galvanic Skin Response (GSR), Heart Rate (HR), Photoplethysmography (PPG). We showed a distribution of emotions for each participant, the baseline set by the iMotion's FACET module and samples outside the first standard deviation (Table 1).

**Result:**

Figure 1 shows a Gaussian or Normal distribution of emotions that validates the number of active participants to be adequate for the study. Joy and Anger were the most frequent states. Figure 2 shows the correlation heatmap illustrating relationships between emotional evidence scores and physiological sensor data. Here, values closer to 1 refer to strong correlation and the sign refers to the relation. Joy showed a weak positive correlation with heart rate (*r* = 0.15, *p* < 0.05), while Fear was moderately correlated with GSR conductance (*r* = 0.35, *p* < 0.01). However, Contempt and Disgust exhibited minimal relationships with physiological data.

**Conclusion:**

Our findings show that there was very little correlation between the facial expression and data captured from wearable sensors. These suggest that traditional statistical modeling may not effectively capture non‐linear dynamics in emotional data. Future research should explore machine learning techniques to better model these complexities and improve emotional detection accuracy.